# Identification of potential biomarkers for abdominal pain in IBS patients by bioinformatics approach

**DOI:** 10.1186/s12876-021-01626-7

**Published:** 2021-02-02

**Authors:** Zhongyuan Lin, Yimin Wang, Shiqing Lin, Decheng Liu, Guohui Mo, Hui Zhang, Yunling Dou

**Affiliations:** 1 Department of Anesthesiology, The First Affiliated Hospital of Sun Yat-sen University, Guangzhou, 510000 Guangdong China; 2 Department of Anesthesiology, Guangdong Second Provincial General Hospital, Guangzhou, 510000 Guangdong China

**Keywords:** Bioinformatics analysis, Biomarker, Visceral hypersensitivity, Abdominal pain, Irritable bowel syndrome

## Abstract

**Background:**

Irritable bowel syndrome (IBS) is the most common functional gastrointestinal disease characterized by chronic abdominal discomfort and pain. The mechanisms of abdominal pain, as a relevant symptom, in IBS are still unclear. We aimed to explore the key genes and neurobiological changes specially involved in abdominal pain in IBS.

**Methods:**

Gene expression data (GSE36701) was downloaded from Gene Expression Omnibus database. Fifty-three rectal mucosa samples from 27 irritable bowel syndrome with diarrhea (IBS-D) patients and 40 samples from 21 healthy volunteers as controls were included. Differentially expressed genes (DEGs) between two groups were identified using the GEO2R online tool. Functional enrichment analysis of DEGs was performed on the DAVID database. Then a protein–protein interaction network was constructed and visualized using STRING database and Cytoscape.

**Results:**

The microarray analysis demonstrated a subset of genes (*CCKBR, CCL13, ACPP, BDKRB2, GRPR, SLC1A2, NPFF, P2RX4, TRPA1, CCKBR, TLX2, MRGPRX3, PAX2, CXCR1*) specially involved in pain transmission. Among these genes, we identified *GRPR, NPFF* and *TRPA1* genes as potential biomarkers for irritating abdominal pain of IBS patients.

**Conclusions:**

Overexpression of certain pain-related genes (*GRPR, NPFF* and *TRPA1*) may contribute to chronic visceral hypersensitivity, therefore be partly responsible for recurrent abdominal pain or discomfort in IBS patients. Several synapses modification and biological process of psychological distress may be risk factors of IBS.

## Background

Chronic abdominal pain is an important symptom of irritable bowel syndrome (IBS), which affects 3.8–9.2% of the global population [1]. Recurrent abdominal pain can substantially reduce quality of life in IBS patients and there is no effective and standard treatment currently [2]. In order to identify new therapies, it is important to understand the underlying mechanism of chronic abdominal pain.

Abdominal pain in IBS is thought to be secondary to visceral hypersensitivity (VH), which has been described as low thresholds of stimuli perception arising from the gut [3]. Visceral hypersensitivity is characterized by two components: allodynia, a painful response to stimuli that are normally not painful and hyperalgesia, an enhanced response to a painful stimulus [4]. The underlying pathogenesis that lead to VH in patients who have IBS is not fully understood. However, the prevailing viewpoint in the pathogenesis involves psychosocial factors, subtle inflammation and alteration in the neuronal excitability of gut sensory pathways, which is responsible for transmitting nociceptive information from the periphery to the central nervous system [5]. Specific changes of a variety of receptors and ion channels both in the expression and function have been documented in visceral pain conditions relevant to irritable bowel syndrome. Researches have shown genetic factors may play a role in those specific changes in IBS [6]. Beyder and colleagues have reported that a mutation of the SCN5A-encoded voltage-gated sodium channel, type V (alpha subunit) was associated with abdominal pain [7]. Other investigators have investigated genetic changes related to enteric infection, epithelial barrier function and immune regulation, serotonin signaling, cannabinoid receptors, and bile acid synthesis, with varying results [8].

Bioinformatics analysis provides tools to produce overviews of genetic networks and potential biological pathways based on large-scale information. This study aimed to explore the molecular mechanisms specially involved in pain transmission underlying IBS and to identify hub genes and potential pathways associated with the pathogenesis of IBS.

## Methods

### Microarray data search and selection of eligible data set

The microarray data of GSE36701 was downloaded from the GEO database (www.ncbi.nlm.nih.gov/geo/) with its microarray platform as GPL570 (Affymetrix Human Genome U133 Plus 2.0 Array). In this microarray, 53 samples of rectal mucosa from 27 IBS-D patients with obvious abdominal pain were obtained and 40 samples from 21 healthy volunteers as controls. Subjects completed a Talley IBS symptom questionnaire modified to include days with pain. IBS was diagnosed according to the Rome II criteria. Those with IBS also completed IBS quality of life questionnaire and the IBS Symptom Severity Score Questionnaire including severity of visceral pain. Those agreeing to take part underwent sigmoidoscopy without bowel preparation. Two biopsies from each donor were obtained using endoscopic biopsy forceps and the mRNA were extracted for further analysis. After RNA extraction and Microarray processing, samples without visible 18S and 28S rRNA peaks were excluded. There was one donor in IBS-D group and two donors in HV group only obtained one microarray data. Details of clinical data collection, RNA extraction and microarray processing can be found in GSE36701 dataset citation (https://www.ncbi.nlm.nih.gov/geo/query/acc.cgi?acc=GSE36701).

### DEGs’ screening

The expression profiles were expressed as FC (fold change) to produce a normally distributed variable. We screened out DEGs using the online tool GEO2R/R, which was based on limma (Linear Models for Microarray Analysis) R package 3.26.8 (https://www.ncbi.nlm.nih.gov/geo/info/geo2r.html). We applied Benjamini and Hochberg false discovery rate method for adjustment of the *P* values. In our study, DEGs between healthy volunteers and IBS-D patients were screened and selected by the cut-off point of FC ≥ 1.2 and *P* value < 0.05.

### Functional enrichment analysis

The selected DEGs were submitted to the Database for Annotation, Visualization, and Integrated Discovery (DAVID) for further analysis. The DAVID database, version 6.8 Beta (https://david-d.ncifcrf.gov/), offers biological function annotation for researchers [9]. In this study, Gene Ontology (GO) analysis and The Kyoto Encyclopedia of Genes and Genomes (KEGG) pathways of DEGs were obtained from the DAVID database. The biological processes and pathways might contribute to verify the significative DEGs and to IBS treatments. Functional annotation analysis was performed by two-sided hypergeometric test and Benjamini and Hochberg methods was applied for multiple testing correction. Min.Count ≥ 2 and *P* value < 0.05 was chosen as the threshold.

### Protein–protein interaction (PPI) analysis

Then we constructed PPI network of DEGs from Search Tool for the Retrieval of Interacting Genes (STRING, http://string-db.org/) [10]. STRING is a public database of known and predicted protein–protein interactions. In this study, we selected confidence score > 0.4 as a threshold to construct the PPI network. The combined score is calculated by combining the probabilities from the different evidence channels, including high throughput experimental data and literature, and corrected for the probability of randomly observing an association [11]. Then, the list of PPI pairs was downloaded for further analysis. Module clustering analysis for the network was then performed to identify the potential functional modules in the network, using the Molecular Complex Detection (MCODE 1.6.1) in Cytoscape 3.7.1 plugin [12, 13]. The degree cut-off value to 2 and the node score cut-off to 0.2 were set as criteria in the MCODE process.

## Results

### Screening and prioritization for DEGs

An available Expression profiling data set (GSE36701) in NCBI was selected in this study to identify differentially expressed genes in the comparisons between IBS-D patients and the healthy volunteers. Then the differentially expressed genes were further filtered for functional associations with neurotransmitters/mediators of pain. Each array was normalized by quantile, and then the DEGs analysis was performed (*P* value < 0.05, FC ≥ 1.2 or ≤ 0.8). Compared with the HVs, we find out 9 upregulated genes (*CCKBR, CCL13, ACPP, BDKRB2, GRPR, SLC1A2, NPFF, P2RX4,* and *TRPA1*) and 4 downregulated genes (*TLX2, MRGPRX3, CXCR1* and *PAX2*) that may have relevance in the initiation and development of visceral pain. The most significant upregulated or downregulated genes specially linked to pain transmission are illustrated in Tables 1 and 2.

**Table 1:** Upregulated genes specially involved in pain transmission in IBS-D patients in comparison with HVs

ID	*P* value	Gene symbol	Gene title	FC
206407_s_at	1.98E−04	CCL13	C–C motif chemokine ligand 13	1.58
205870_at	1.51E−03	BDKRB2	Bradykinin receptor B2	1.38
217590_s_at	1.62E−02	TRPA1	Transient receptor potential cation channel subfamily A member 1	1.23
204393_s_at	4.26E−03	ACPP	Acid phosphatase, prostate	1.55
208389_s_at	1.11E−02	SLC1A2	Solute carrier family 1 member 2	1.31
204088_at	1.41E−02	P2RX4	Purinergic receptor P2X4	1.23
207929_at	2.62E−02	GRPR	Gastrin releasing peptide receptor	1.37
206402_s_at	4.65E−02	NPFF	Neuropeptide FF-amide peptide precursor	1.25
234475_x_at	1.70E−02	CCKBR	Cholecystokinin B receptor	1.77

*HVs* healthy volunteers, *FC* fold change

**Table 2:** Downregulated genes specially involved in pain transmission in IBS-D patients in comparison with HV

ID	*P* value	Gene symbol	Gene title	FC
207410_s_at	2.27E−02	TLX2	T-cell leukemia homeobox 2	0.65
1553293_at	1.24E−02	MRGPRX3	MAS related GPR family member X3	0.71
206228_at	2.58E−02	PAX2	Paired box 2	0.76
207094_at	7.11E−03	CXCR1	C-X-C motif chemokine receptor 1	0.57

*HVs* healthy volunteers, *FC* fold change

### Functional enrichment analysis

**Fig. 1:**
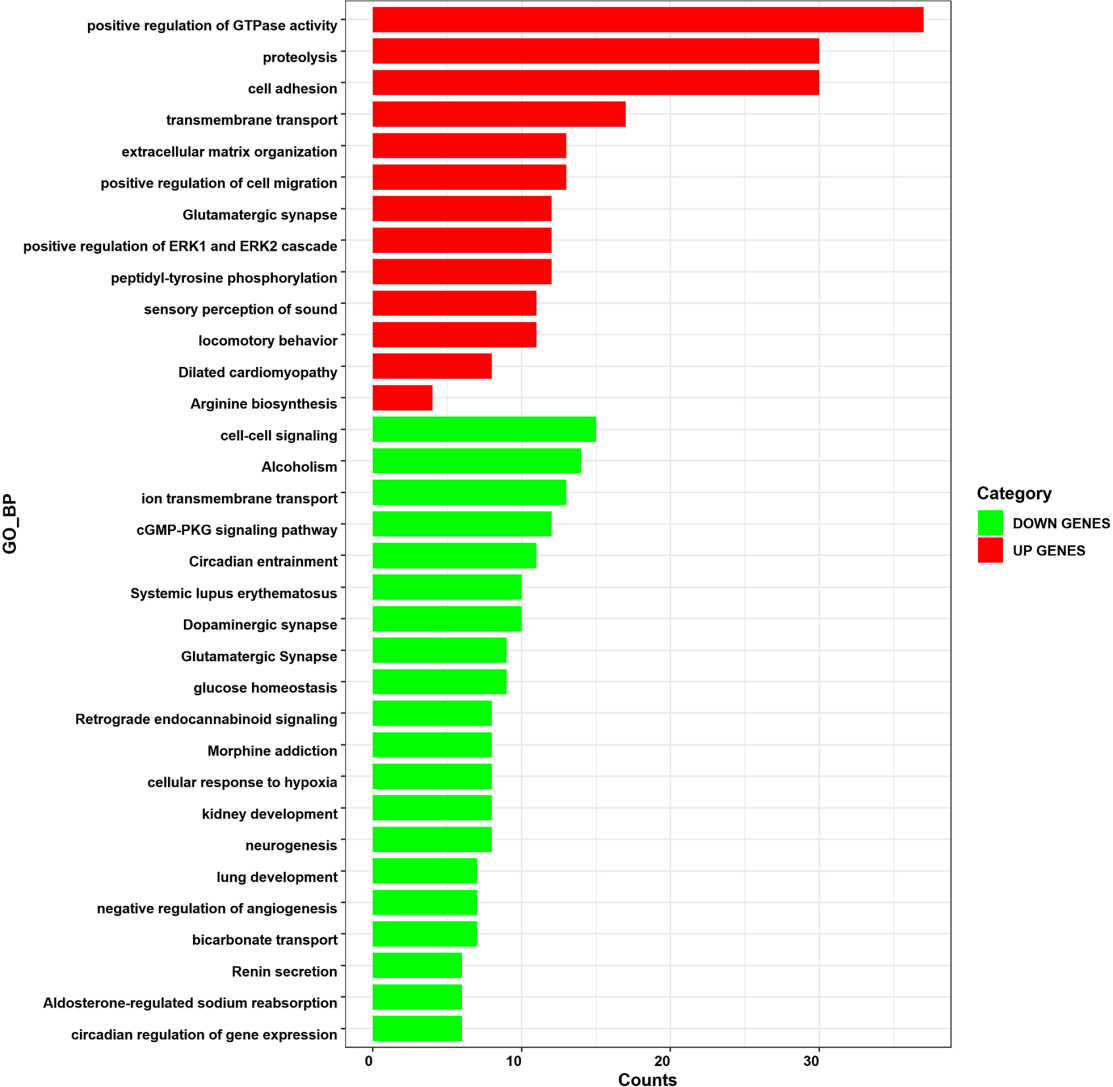
Kyoto Encyclopedia of Genes and Genomes (KEGG) pathways enriched by the differentially expressed genes (DEGs). The x-axis of graph shows the counts, the number of genes clustered in each category, increasing with bar length. The threshold of *P* value was set as 0.05

**Fig. 2:**
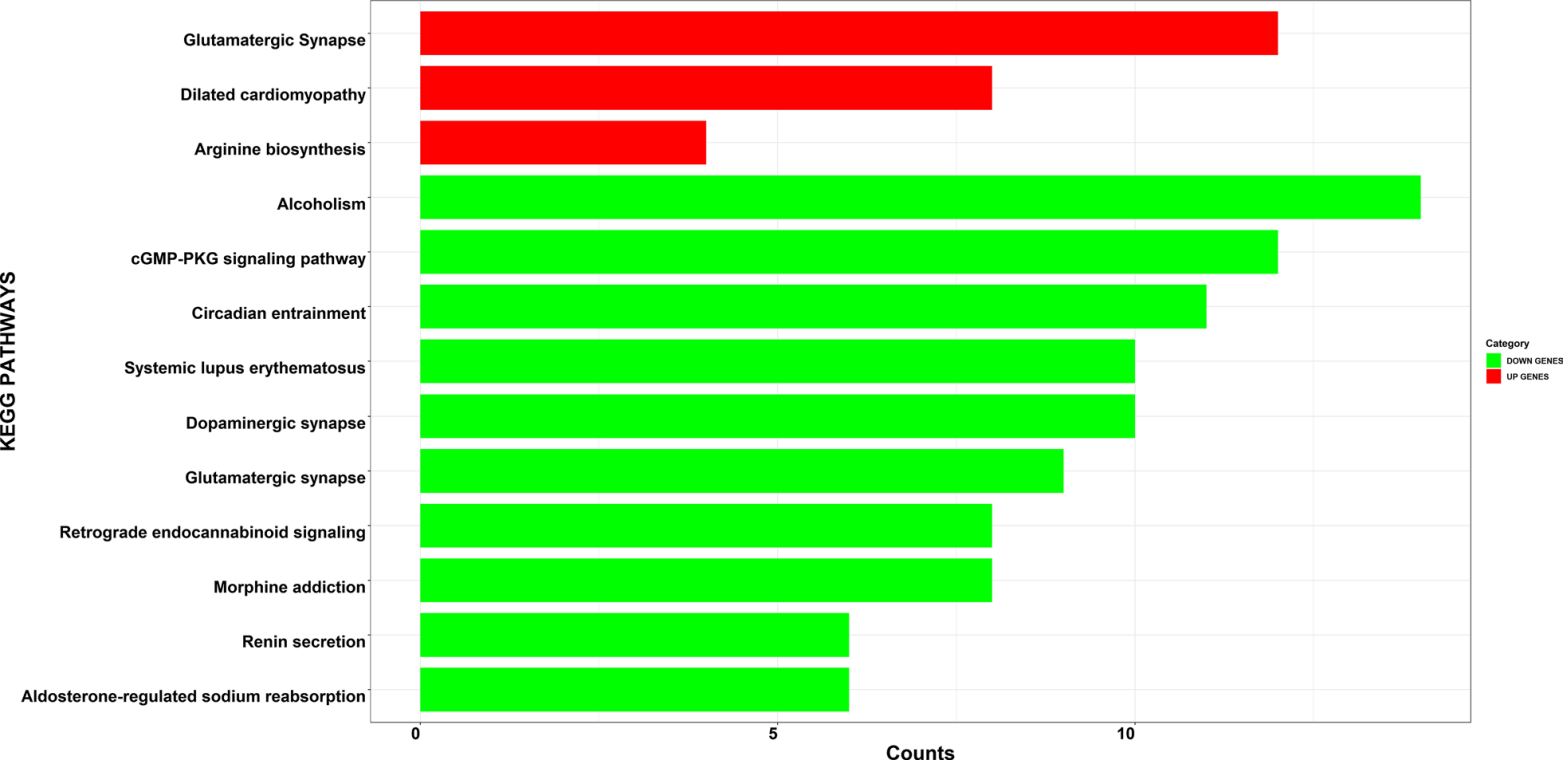
Biological process (BP) enriched by the differentially expressed genes (DEGs). The x-axis of graph shows the counts, the number of genes clustered in each category, increasing with bar length. The threshold of *P* value was set as 0.05

Functional analysis was carried out with GO biological process and KEGG pathways. Genes differentially were classified according to their putative Gene Ontology (GO) based on similarity with known genes recorded in public databases (Fig. 1). To further understand the functions of the DEGs, we represented significant pathways based on KEGG databases (Fig. 2). The upregulated DEGs were mainly associated with Glutamatergic synapse, Dilated cardiomyopathy, Arginine biosynthesis. The downregulated DEGs were linked to Circadian entrainment, Alcoholism, Aldosterone-regulated sodium reabsorption, Maturity onset diabetes of the young, cGMP-PKG signaling pathway, Dopaminergic synapse, Morphine addiction, Systemic lupus erythematosus, Glutamatergic synapse, Glycine, serine and threonine metabolism, Retrograde endocannabinoid signaling, Renin secretion.

### PPI network

**Fig. 3:**
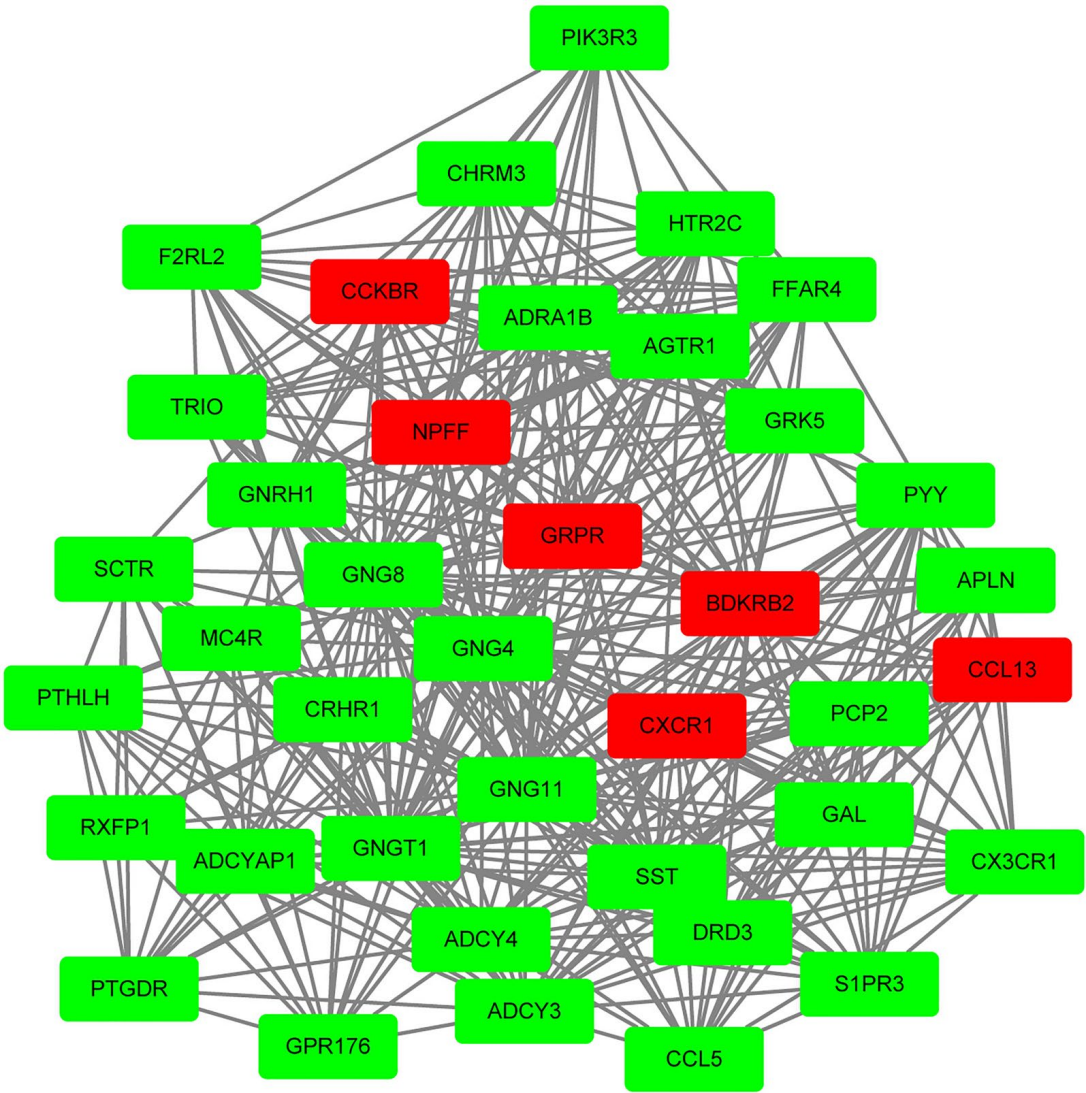
The Molecular Complex Detection (MCODE) components of the protein–protein interaction (PPI) network identified gens associated with pain transmission. Several outstanding hub genes such as *CCKBR, NPFF, GRPR, BDKRB2, CXCR1* and *CCL13* (red rectangle) was clustered. Degree > 10 was set as the cut-off criterion. Rectangle represent DEGs and lines represent interaction and relationship between nodes

A PPI network of the proteins encoded by identified DEGs was constructed by STRING. To identify the key modules of the PPI network, module clustering was then performed through the MCODE plugin of Cytoscape (Fig. 3). The modules showed that several pain or itch related genes like *GRPR, NPFF, CCKBR, BDKRB2, CXCR1* and *CCL13* genes (Red Rectangle, Fig. 3) were also clustered and most of these genes also occurred in GO terms or KEGG pathways enriched above. Finally, we identified that *GRPR, NPFF* and *TRPA1* as potential biomarkers for abdominal pain of IBS patients.

## Discussion

Persistent hypersensitivity of sensory pathways innervating the colon is regarded as primary mechanism contributing to the initiation, development, and maintenance of chronic discomfort and abdominal pain in IBS patients (15, 16, 39). Therefore, determining the mechanisms contributing to these processes is crucial. Swan et al. originally submitted the GSE36701 dataset, performed mRNA expression profiling study of rectal biopsies from donors with healthy volunteers and IBS-D patients [14]. They collected detailed clinical data (including abdominal pain frequency, anxiety and depression scale), using a combination of bioinformatics and experimental approaches to identify candidate genetic polymorphisms in the IBS [14]. While we further filtered for functional associations related to neurotransmitters/mediators of pain. Most notable among those genes were shown in Table 1 (upregulated genes) and Table 2 (downregulated genes). Interestingly, *GRPR, NPFF, TRPA1, BDKRB2, MRGPRX3*, that commonly regarded as factor also regulating itch signaling pathways were found [15–17]. Finally, three genes including *GRPR, NPFF* and *TRPA1* were considered to play an essential role in abdominal pain in IBS.

In the colon, afferent sensitization occurs via a variety of processes [18], including histamine-dependent mechanisms and histamine-independent mechanisms [19, 20]; Evidence have been accumulated that activation of receptors associated with the above two itch pathways on colon-innervating afferents induces visceral hypersensitivity [19, 21]. Gastrin-releasing peptide receptor expressing (GRPR)+ neurons have a central role in the spinal transmission of both histaminergic and non-histaminergic itch [22]. In our study, *GRPR* was significantly upregulated in IBS-D patients and identified as a hub gene by MCODE. GRPR is a G protein-coupled receptor and mediates itch sensation mainly via the PI3Kγ/Akt pathway [23]. At the same time, GRP induces neutrophil chemotaxis through GRPR via p38, ERK1/2 [24] and PI3K activation and p38, ERK and PI3K are important mediators in visceral pain [25–27]. Itch and pain signals are conveyed by distinct yet interacting neuronal pathways: pruritogens at higher doses produce pain to suppress itch [28, 29]; the frequency of abdominal pain in IBS was higher in patients with chronic pruritus than in healthy controls [30]. In addition, Neuropeptide FF (*NPFF*), the histamine-independent itch receptors agonist, evoking colonic afferent mechanical hypersensitivity [17, 19], was also clustered by Cytoscape module in our study. Therefore, we speculated that altered GRP-GRPR signaling and *NPFF* in spinal dorsal horn participate in visceral hypersensitivity through sensitization of itch transmission neurons, thereby contributing to abdominal pain or discomfort.

In the present study, another gene related to pain transmission deserve attention. Our results, together with previous studies have indicated significantly up-regulated *TRPA1 *(Transient receptor potential ankyrin 1) mRNA expression in biopsies of IBS patients [31]. TRPA1 has been implicated in mechanical hypersensitivity of colonic afferents and both bradykinin and TNF-α induce visceral hypersensitivity through a TRPA1-dependent mechanism [32]. On the other hand, TRPA1 can also induce the inflammatory response via neurogenic inflammation: activation and sensitization of TRPA1 and release of substance P contribute to the initiation and development of colitis in mice, which correspondingly re-sensitises nociceptors [33]. TRPA1 expressed by intestinal enterochromaffin cells can serves as the primary detector of intestinal irritants prior to direct sub-mucosal damage [34]. These findings further highlight TRPA1 as an important integrator of sensory signals in colonic afferents by inducing mechanical visceral hypersensitivity.

As we known, IBS is a multifactorial disease. Abnormal stress response, psychological distress and infectious or inflammatory response in susceptible population may initiate visceral hypersensitivity that results in the development of IBS symptoms. According to the functional enrichment analyses, two pathways were associated with dysregulation of renin-angiotensin system (RAS), which is a potent target of stress-induced intestinal inflammation in a murine model of IBS [35]. Circadian entrainment and disorder of several synapses such as Glutamatergic synapse, Dopaminergic synapse may be risk factors of IBS. Besides, certain pathways directly involved in pain transmission were enriched, including Morphine addiction and Retrograde endocannabinoid signaling. Endocannabinoids are involved in controlling motility, secretion and intestinal inflammation [36]. The endocannabinoid system in DRG neurons mediate stress-induced visceral hypersensitivity in a mouse model of IBS [37].

## Conclusions

Our results showed that *GRPR, NPFF* and *TRPA1* genes may be potential biomarkers for the diagnosis and new targets for treatment of abdominal pain in IBS. Several synapses modification and biological process of psychological distress may be risk factors of IBS. However, further studies are required to confirm the clinical significance of these findings.

## Data Availability

The raw data of microarray experiment (its accession numbers are GSE36701) was downloaded from the Gene Expression omnibus (GEO) repository (https://www.ncbi.nlm.nih.gov/geo/). All data are publicly accessible. All data generated or analyzed during this study are included in this published article.

## Abbreviations

IBS: Irritable bowel syndrome
HV: Healthy volunteer
GRPR: Gastrin-releasing peptide receptor
VH: Visceral hypersensitivity
TRPA1: Transient receptor potential ankyrin 1
NPFF: Neuropeptide FF
GEO: Gene expression omnibus
DEGs: Differentially expressed genes
IBS-D: Irritable bowel syndrome with diarrhea
DAVID: Database for annotation, visualization, and integrated discovery
STRING: Search tool for the retrieval of interacting genes
GO: Gene ontology
KEGG: Kyoto encyclopedia of genes and genomes
PPI: Protein–protein interaction
MCODE: Molecular complex detection
CXCL13: C-X-C motif chemokine ligand 13
CXCR1: C-X-C motif chemokine receptor 1
